# A structural backbone with sequestered plasticity organizes the *Escherichia coli* pangenome

**DOI:** 10.1128/msystems.00207-26

**Published:** 2026-06-15

**Authors:** Yi-Fei Lu, Guang-Hong Zuo, Xiao-Yang Zhi

**Affiliations:** 1 Key Laboratory of Microbial Diversity in Southwest China of Ministry of Education, School of Life Sciences, Yunnan Institute of Microbiology, Yunnan University 12635, Kunming, China; 2 Wenzhou Institute, University of Chinese Academy of Sciences, Wenzhou, China; Universidad Miguel Hernandez de Elche, San Juan de Alicante, Alicante, Spain

**Keywords:** pangenome, alignment-free, orthology inference, gene synteny network, genomic architecture, *Escherichia coli*, genomic islands

## Abstract

The exponential growth of microbial genomic data has made computational scalability the primary bottleneck in pangenome analysis because traditional alignment-based methods have quadratic complexity. We developed CVNet, an alignment-free orthology inference framework that uses composition vectors and Markov clustering. CVNet achieves near-linear scalability and high accuracy, enabling pangenome analysis across thousands of genomes. Applying it to 1,200 complete *Escherichia coli* genomes, we moved beyond the “bag-of-genes” approach to investigate the pangenome’s spatial architecture. By constructing a genome-wide core gene synteny network from CVNet orthogroups, we revealed that core genes form a modular (*Q* = 0.9851) and asymmetric structural backbone, strongly biased toward the replication origin (*oriC*; KS test, *D* = 0.9133). Accessory genes and genomic islands are non-randomly sequestered within specific integration hotspots, with over 99% located between core genes connected by extremely weak syntenic links. These findings establish a structural backbone and sequestered plasticity model, demonstrating how *E. coli* maintains chromosomal integrity through a rigid scaffold that physically compartmentalizes genetic plasticity. This study thus presents CVNet as a scalable computational solution and introduces a spatial paradigm for understanding how bacterial genomes balance evolutionary stability with adaptive flexibility.

**IMPORTANCE:**

Pangenome analysis has been constrained by alignment-based tools that do not scale and a “bag of genes” perspective that ignores chromosomal organization. We present CVNet, an alignment-free framework that enables near-linear scalability for orthology inference across thousands of genomes. Applying CVNet to 1,200 complete *E. coli* genomes, we discover that the chromosome is organized by a rigid core gene backbone, with accessory genes sequestered into discrete integration hotspots. This structural backbone and sequestered plasticity model reveals bacterial genomes as spatially organized systems in which stability and flexibility are physically compartmentalized, thereby establishing a framework for topological pangenomics.

## INTRODUCTION

The rapid advancement of high-throughput sequencing technologies has catalyzed the transition of microbial research from single-strain analysis to population genomics (1, 2). In this field, pangenomics has emerged as a powerful framework for elucidating the mechanisms underlying microbial speciation and adaptive evolution (3–6). By systematically partitioning the total gene repertoire into conserved, accessory, and rare components, this approach reconstructs a population’s evolutionary trajectory and maps the structural blueprint underlying both its genomic stability and adaptive potential (7). The bacterial pangenome thus represents a complex hierarchy of gene families categorized by conservation levels: the core genome, comprising genes nearly universal across all members of a species and essential for fundamental biology; the shell genome, which includes genes present at intermediate frequencies and often drives lineage-specific differentiation; and the cloud genome, representing rare sequences frequently associated with transient environmental adaptation (8). This continuous spectrum of gene occupancy not only mirrors the composite selective pressures acting on a population but also provides the foundation for investigating how genomic architecture constrains or facilitates adaptation (9–11).

Because pan-genome studies typically encompass hundreds or even thousands of genomes, the sheer volume of pairwise sequence alignments required has pushed traditional analytical frameworks to their limits, making computational scalability a core bottleneck. This bottleneck primarily stems from the traditional paradigm of all-versus-all, alignment-dependent orthology inference, in which establishing homology requires pairwise sequence comparisons that scale with the square of the data set size (12–14). To circumvent the all-versus-all alignment bottleneck, three principal strategies have emerged: (i) sequence-space reduction, as implemented in Roary (15), PIRATE (16), PEPPAN (14), and PanTA (12) via gene sequence pre-clustering and PGAP2 (17) via whole genome pre-clustering; (ii) incremental orthology assignment, where OrthoFinder v3.0 builds upon a core set of genomes (18); and (iii) comparative screening via machine learning, a method pioneered by SonicParanoid2 to skip improbable comparisons (19). Underpinning these strategies, the foundational sequence-search algorithms have undergone a dramatic speed increase. The shift from exhaustive BLAST (20) to heuristics-based DIAMOND (21), and further to ultra-fast, *k*-mer-indexed MMseqs2 (22), has dramatically accelerated the core step of homology detection.

Despite these optimizations at both the workflow and algorithmic levels, a fundamental bottleneck persists. These approaches remain tethered to the alignment paradigm; even *k*-mer-based pre-filters, such as those in MMseqs2, merely accelerate the alignment process rather than replacing it as the primary means of homology inference. Consequently, the indispensable alignment, coupled with its nonlinearly growing demand, causes the overall time complexity to escalate uncontrollably as the data set size increases. This computational bottleneck inevitably forces a pragmatic compromise: researchers must choose between analyzing thousands of genomes and maintaining the resolution needed to detect subtle or rare evolutionary events. As a result, population-scale analyses risk overlooking critical signals, including recent horizontal gene transfers (HGTs) and fine-scale adaptive signatures. Therefore, to fully harness the potential of massive genomic data sets without this trade-off, a paradigm shift may be necessary (23–25). We argue that the next leap forward requires truly alignment-free methodologies to enable scalable, high-resolution orthology inference and unlock previously inaccessible dimensions of population genomics.

In prokaryotes, most large-scale analyses still treat the pangenome as a bag of genes, focusing primarily on the presence-absence variation of orthogroups while largely ignoring their physical arrangement on the chromosome (26). This oversight has, until recently, been a practical necessity; the predominance of fragmented draft genomes in public databases has precluded reliable reconstruction of genome-wide gene order (synteny). Although synteny is an established criterion for refining orthology assignments in genomic comparisons (27–29), its utility has been constrained by the draft status of most genomes, which limits analysis to local gene-order conservation. Consequently, our understanding of bacterial genomic architecture—specifically, how evolutionarily stable core elements and dynamically acquired accessory elements are structurally integrated—remains strikingly limited. The genomic architecture defined by synteny is a direct and important record of evolutionary forces (30, 31). Tightly conserved core gene blocks are thought to reflect purifying selection that may support functional synergy, transcriptional coordination, and the maintenance of essential chromosomal structure (32–34). In contrast, the variable positioning of accessory genes reveals hotspots of genomic flux, where relaxed structural constraints permit horizontal gene transfer, recombination, and the integration of genomic islands (GIs) (35–37). Thus, the spatial interplay between core and accessory regions reflects the fundamental tension between evolutionary stability and adaptive flexibility in bacterial genomes.

Several high-quality pangenome studies of *E. coli* have been conducted with alignment-based tools. For instance, Yang and colleagues analyzed 491 complete genomes with Roary, identifying 867 core genes and revealing host-specific accessory gene signatures (38). More recently, Chauhan et al. analyzed 2,377 complete genomes using non-negative matrix factorization (NMF), revealing a structured pangenome matrix (2,398 core, 5,182 accessory, and 163,619 rare genes) that delineated 31 co-occurrence modules (39). While these studies provided valuable insights into gene content variation, host adaptation, and the mathematical structure of the pangenome, they treated the pangenome primarily as a collection of independent gene families (or their presence/absence patterns), without considering the linear order of genes along the chromosome. As a result, the spatial architecture—how core and accessory genes are physically organized and integrated into a stable yet flexible chromosomal framework—remains unexplored. Fortunately, the ongoing revolution in long-read sequencing is rapidly dismantling the data barrier (40). The growing availability of high-quality complete genomes now makes it feasible to move beyond simple gene inventories and to rigorously interrogate the evolutionary constraints and functional implications that govern the physical organization of bacterial chromosomes.

To overcome the computational bottleneck inherent in alignment-based orthology inference, we developed CVNet, an alignment-free framework for high-throughput analysis. CVNet represents genes as composition vectors (CVs) and uses Markov-based background correction, avoiding exhaustive sequence alignments to enable rapid and accurate clustering of orthogroups across thousands of genomes. With this scalable tool in hand, we then turned to the unresolved conceptual question of pangenome spatial architecture. We applied CVNet to a meticulously curated data set of 1,200 complete *Escherichia coli* genomes. Leveraging the high-resolution orthogroups identified, we constructed a core gene synteny network—a dedicated topological analysis—to decode the principles governing the spatial organization of the chromosome. *E. coli*, with its open pangenome and frequent horizontal gene transfer, provides an ideal yet rigorous testbed for investigating whether a stable genomic architecture persists amid substantial sequence flux. Our analysis reveals that the chromosome is organized around a highly modular, asymmetric structural backbone composed of core genes. Accessory genetic elements, including genomic islands, are not randomly dispersed but are precisely sequestered within defined integration hotspots, which are characterized by weak syntenic linkages between core gene blocks. This “structural backbone and sequestered plasticity” model shows how combining scalable orthology inference with subsequent spatial analysis can resolve the long-standing question of how bacterial genomes balance evolutionary stability with adaptive flexibility. Thus, this study makes a dual contribution: it introduces CVNet as an efficient and scalable tool that redefines the feasibility of large-scale pangenomics, and it provides a novel, spatially resolved perspective on the genomic architecture that underpins the evolutionary cohesion of a major bacterial species.

## RESULTS

### Overview of CVNet workflow: from composition vectors to orthogroup identification

**Fig 1:**
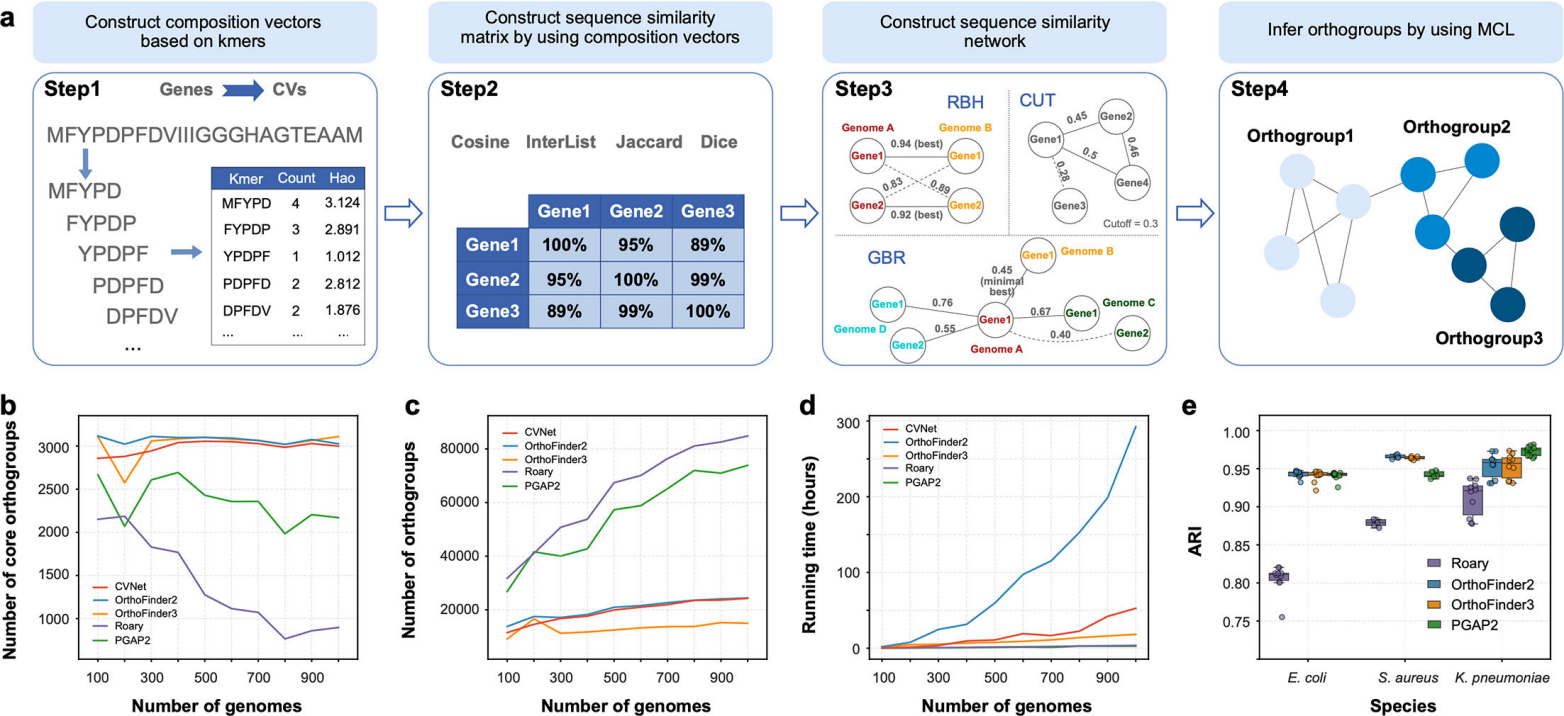
CVNet workflow and performance benchmarking. (**a**) Overview of the CVNet pipeline. The process consists of four stages: (1) *k*-mer decomposition and composition vector (CV) construction; (2) similarity matrix calculation using multiple metrics; (3) gene association network construction via RBH, CUT, or GBR strategies; and (4) orthogroup identification using the Markov clustering (MCL) algorithm. (**b**) Stability of core gene identification. Comparison of core genome size as a function of the number of genomes among CVNet, OrthoFinder2, OrthoFinder3, Roary, and PGAP2. (**c**) Pangenome expansion analysis. (**d**) Computational efficiency. Comparison of running time (CPU hours) across different data sets. (**e**) Inference accuracy. Performance evaluation using the adjusted rand index (ARI) across multiple bacterial species.

CVNet employs an alignment-free framework that partitions genes into evolutionary units using a four-stage pipeline (Fig. 1a). In step 1, the workflow processes raw protein (FAA) or coding nucleotide sequences (FFN) by decomposing them into short, fixed-length strings (*k*-mers). For instance, a protein sequence is scanned to count occurrences of overlapping peptides (e.g., MFYPD and FYPDP). To refine these high-dimensional CVs, users can choose the Count method for raw frequency analysis or the Hao method (41), which applies background correction with a Markov model to suppress stochastic noise from neutral mutations. In step 2, CVNet quantifies relationships between gene pairs by computing a comprehensive similarity matrix. This is done using a suite of metrics, including Cosine similarity, InterList similarity, Jaccard index, and Dice coefficient, providing flexibility across different biological contexts. Proceeding to step 3, this matrix is transformed into a gene association network using three distinct edge-formation strategies: reciprocal best hit (RBH), which identifies mutually highest-scoring pairs (e.g., similarity scores of 0.94 and 0.92) for high-confidence ortholog inference; cut-off (CUT), which connects all pairs exceeding a predefined threshold (e.g., 0.3); and global best reciprocal (GBR), a hybrid approach that sets a global threshold as the minimum value among all reciprocal best hits (e.g., 0.45) to capture additional putative relationships. Finally, in step 4, the Markov clustering (MCL) algorithm is applied to the resulting network (42). By simulating stochastic flow through iterative expansion and inflation steps, MCL partitions the complex network into discrete, densely interconnected subgraphs. These subgraphs constitute the final predicted orthogroups (e.g., orthogroups 1–3), representing sets of evolutionarily linked and functionally synchronized genes.

### Performance of CVNet: scalability, stability, and accuracy

To evaluate the robustness and broad applicability of CVNet, we conducted extensive benchmarking using *Escherichia coli* as the primary model (Table S1), complemented by accuracy assessments across multiple bacterial species. CVNet was compared with several state-of-the-art tools, including OrthoFinder2, OrthoFinder3, Roary, and PGAP2. All tools were run with their default parameters. The results showed that as the number of genomes increased, the core gene counts predicted by most methods decreased; notably, CVNet and OrthoFinder remained within a stable range, with CVNet maintaining a highly consistent core genome of approximately 3,000 genes (Fig. 1b). Remarkably, this core size aligns with data from multiple early 2-D gel electrophoresis experiments on *E. coli*, which estimated approximately 3,000 detectable proteins (43–45). This core genome size (3,053 orthogroups) is substantially larger than the 867 core genes reported for 491 genomes (38) and than the result reported for 2,377 complete genomes (39). The difference reflects both the expanded data set (1,200 vs. 491 genomes) and CVNet’s ability to capture more complete orthologous relationships through its alignment-free, composition-based clustering, which avoids overly stringent filtering that can arise from pairwise alignment thresholds. Notwithstanding Chauhan et al.’s larger genome cohort, the distinctions between our respective results are highly nuanced. Chauhan et al. employed NMF on a gene presence/absence matrix to define core genes via a mathematically determined cutoff (a 6.8% absence frequency). In contrast, CVNet applies a strict ≥99% frequency threshold to core orthogroups and uses Markov clustering based on composition-vector similarity. This methodology tends to generate more inclusive orthogroups by tolerating moderate sequence divergence, which traditional alignment-based pipelines might otherwise segregate into distinct families. Consequently, CVNet retains more gene families as “core” than NMF may classify as accessory or rare because of stricter presence/absence cutoffs or differences in orthogroup granularity.

Theoretically, a species’ core gene set should remain relatively stable regardless of the number of genomes analyzed; this stability underscores CVNet’s robust orthology inference capability. Regarding pangenome expansion, although the number of non-core genes increased with the addition of genomes across all tools, PGAP2 and Roary showed a significantly higher rate of increase than CVNet and OrthoFinder (Fig. 1c). The pan-genomic growth of all methods demonstrated varying degrees of growth trends, confirming that *E. coli* possesses an open pan-genome, which is consistent with previous research findings (46–48). However, the divergent growth trajectories also highlight distinct approaches to sequence homology analysis. CVNet and OrthoFinder both employ MCL clustering, producing more aggressive clustering within orthogroups, whereas Roary and PGAP2 adopt a more conservative strategy. Additionally, we investigated the impact of various algorithmic parameters, such as similarity and edge-construction methods, on clustering results to optimize benchmarking performance (Table S2). To assess computational efficiency, we tested the tools on 10 data sets ranging from 100 to 1,000 genomes. CVNet demonstrated near-linear scalability. Although PGAP2 and Roary were faster due to their pre-clustering heuristics, CVNet showed a substantial efficiency gain over traditional all-to-all approaches, running approximately six times faster than OrthoFinder2 (Fig. 1d). Furthermore, we evaluated inference accuracy across multiple species, including *E. coli*, *Staphylococcus aureus* (Table S3), and *Klebsiella pneumoniae* (Table S4). CVNet consistently achieved superior performance with ARI exceeding 0.95 (Fig. 1e), validating its reliability across diverse bacterial taxa.

### Pangenome structure and core gene distribution in 1,200 complete *Escherichia coli* genomes

**Fig 2:**
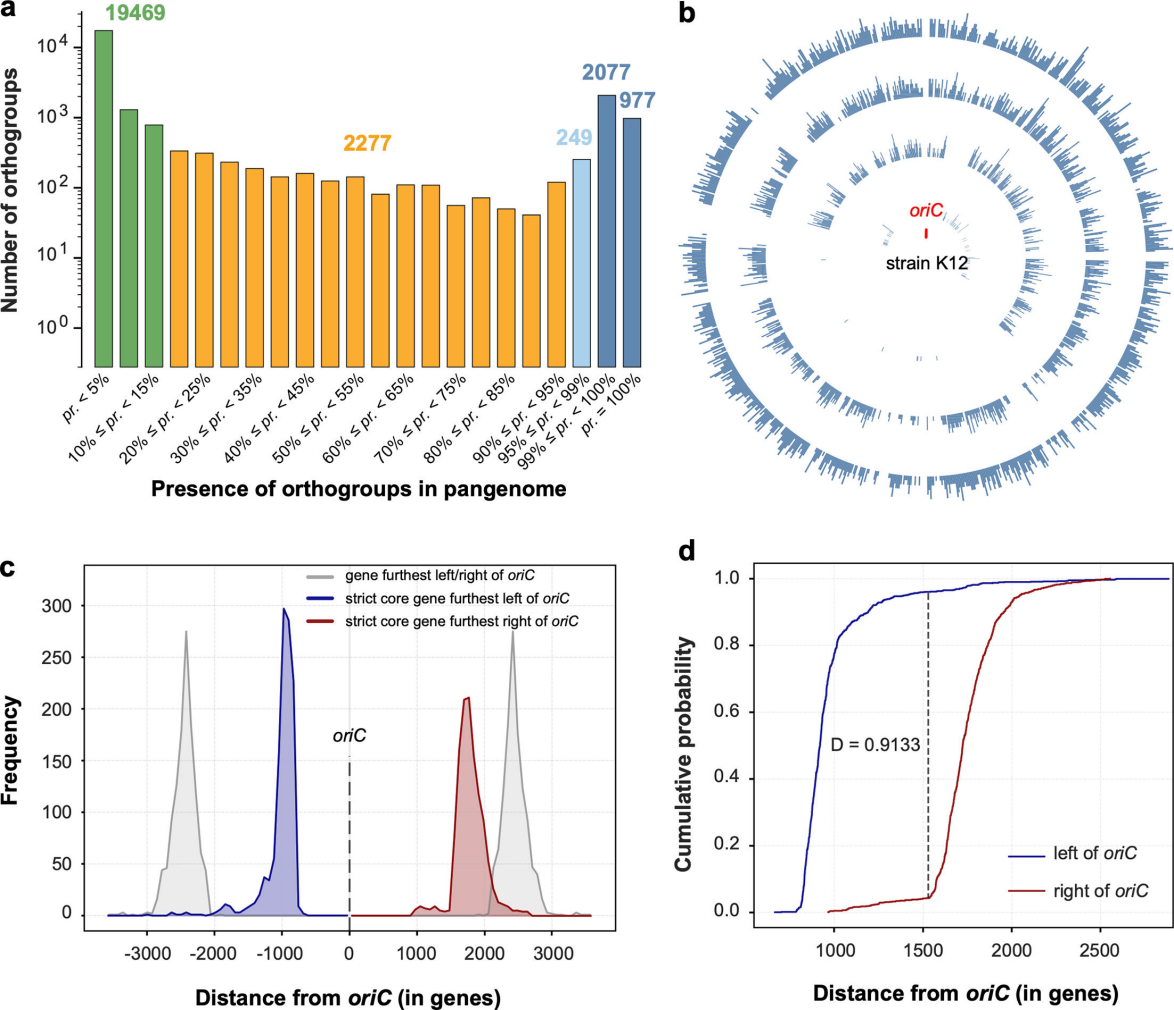
Pangenome structure and spatial distribution of 1,200 *Escherichia coli* genomes. (**a**) Pangenome composition. Distribution of orthogroups classified as core, softcore, shell, and cloud based on their occupancy across 1,200 genomes. (**b**) Spatial distribution of core genes. Genomic positions of core orthogroups relative to the replication origin (*oriC*) in the reference strain *E. coli* K12. (**c**) Asymmetric distribution across the population. Density plot showing the consistent positional bias of core genes across all 1,200 strains. (**d**) Statistical significance of positional bias. Cumulative distribution function (CDF) and Kolmogorov-Smirnov (KS) test results confirming the non-random organization of the core genome (*D* = 0.9133).

To demonstrate the robustness and scalability of CVNet on large-scale genomic data sets, we conducted a comprehensive pangenome analysis of 1,200 complete *E. coli* genomes. Using CVNet, we identified 25,049 orthogroups, revealing a characteristic pangenome structure: 3,053 core orthogroups (13.98%), 249 softcore orthogroups (0.99%), 2,277 shell orthogroups (9.09%), and 19,469 cloud orthogroups (77.72%; Fig. 2a). This distribution highlights the extensive genetic diversity within the *E. coli* species. The core genome size aligns with previous results across multiple data sets and bioinformatics tools (17, 47, 49), underscoring CVNet’s precision in partitioning pangenome components from large-scale sequence data. We further investigated the spatial organization of these core genes on the bacterial chromosome. Using the *E. coli* strain K12 as a reference, our analysis revealed that strict core genes are primarily clustered near the replication origin (*oriC*), likely reflecting evolutionary constraints that maintain essential biological functions and maximize gene dosage effects (50, 51). However, their distribution on either side of the *oriC* is markedly asymmetric (Fig. 2b). To determine whether this observation reflects a general organizational principle, we extended this spatial analysis across the entire collection of 1,200 genomes. The results confirmed that this asymmetric bias is highly consistent across diverse strains, with core genes showing a significant and reproducible distributional skew relative to the *oriC* (Fig. 2c). A Kolmogorov-Smirnov test was used to quantify this spatial divergence, yielding a substantial effect size of *D* = 0.9133 (Fig. 2d). This robust statistical evidence confirms that the arrangement of core genes is significantly non-random and asymmetric, suggesting that although core genes are anchored near the *oriC* for stability, their precise positioning is subject to distinct selective pressures or chromosomal structural constraints. This asymmetric bias is consistent with a recent large-scale study analyzing 910 bacterial species, which found that approximately two-thirds (65.8%) of gene families are subject to natural selection at specific chromosomal positions (52). They demonstrated that this selection is primarily driven by growth-rate-dependent gene dosage effects: during rapid growth, genes near the replication origin (*oriC*) have higher relative copy numbers than those near the terminus (*ter*), conferring a fitness advantage to origin-proximal positioning.

To assess the robustness of the identified orthogroups, we quantified the intra-group sequence similarity and its variance for each OG. A scatter plot of these two metrics (Fig. S1) shows that the vast majority of OGs maintain high internal sequence consistency, as indicated by elevated similarity scores and minimal variance, underscoring CVNet’s high precision in clustering evolutionarily conserved sequences. Building upon this high-quality clustering, we partitioned the pangenome into core and non-core categories using a 99% frequency threshold and performed functional annotation using COG classifications (Fig. S2). Our analysis revealed a clear functional dichotomy: core genes are significantly enriched in categories essential for fundamental biological maintenance, including J (Translation, ribosomal structure, and biogenesis), E (Amino acid transport and metabolism), and F (Nucleotide transport and metabolism). Conversely, non-core genes show greater enrichment in categories linked to environmental adaptation and genomic plasticity, including L (Replication, recombination, and repair), M (cell wall/membrane/envelope biogenesis), and K (Transcription). These findings suggest that while the core genome maintains the stability of primary metabolism, the non-core genome provides the regulatory and structural diversity that enables *E. coli* to thrive across diverse ecological niches.

### Topological landscape and community architecture of the core gene synteny network

**Fig 3:**
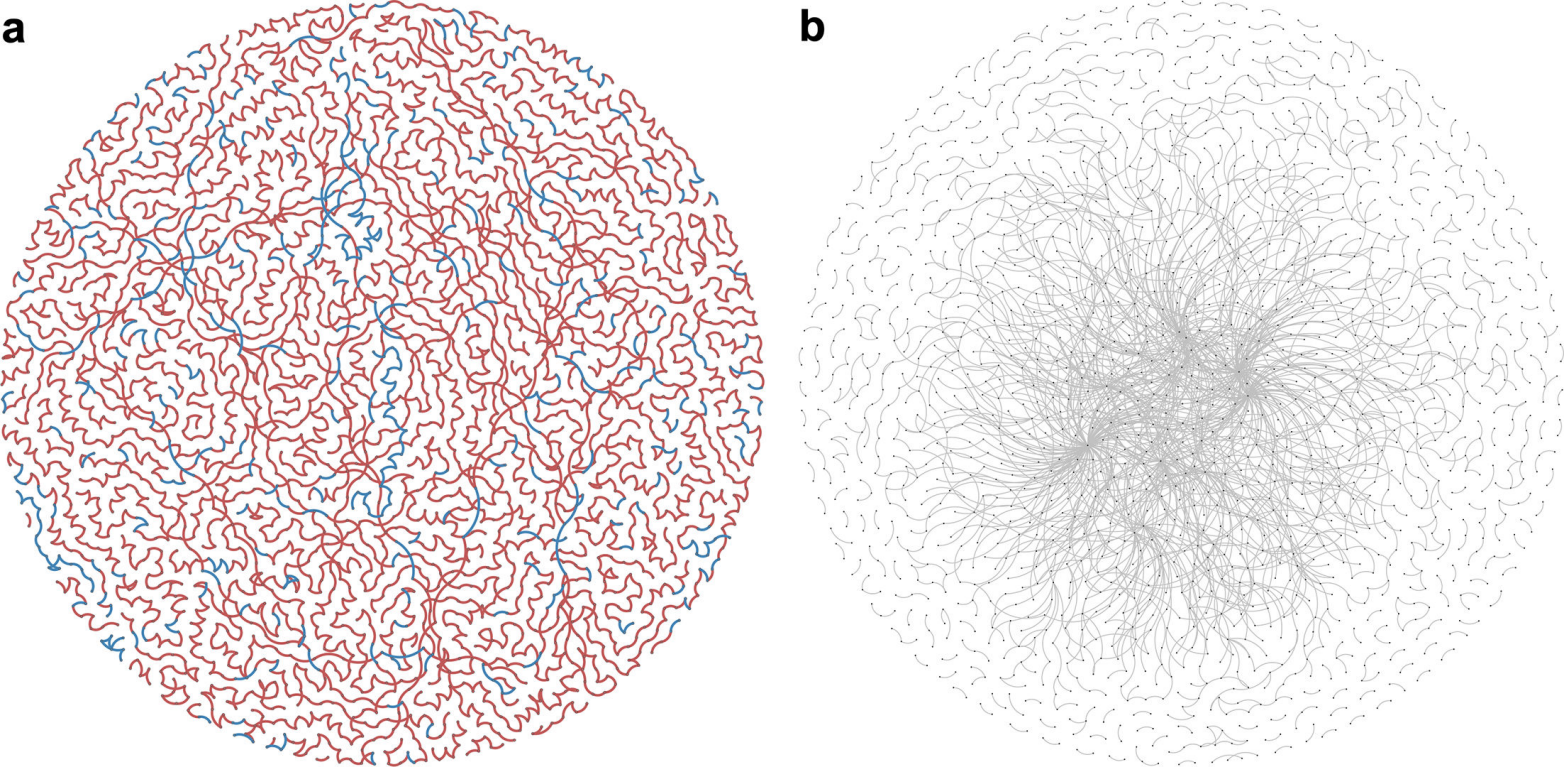
Core orthogroup synteny network and connectivity patterns. (**a**) Global synteny network. Visualization of the association network where nodes represent core orthogroups, and edges represent spatial adjacency. Edge colors indicate weights, with red representing high stability (weight >0.9) and blue representing low-frequency co-occurrence (0.01 < weight < 0.9). (**b**) Characterization of low-weight edges. Detailed view focusing on edges with weights below 0.01. These sparse connections capture transient co-occurrences and genomic rearrangements, highlighting the flexible segments within the otherwise stable core structural backbone.

To further explore the conserved spatial organization of the core genome, we constructed a comprehensive synteny network based on the chromosomal adjacency of 3,053 core orthogroups across 1,200 complete *E. coli* genomes. In this network, nodes represent individual core orthogroups, and edges denote the frequency (weight) with which two core OGs appear as immediate neighbors on the chromosome. Based on the edge weights, the network exhibited two distinct connectivity patterns. In Fig. 3a, which focuses on edges with weights exceeding 0.01, a clear hierarchy emerges: high-weight connections (red, weight >0.9) form a dense, tightly interconnected subnetwork, indicating gene pairs that maintain extreme spatial stability across the vast majority of genomes. In contrast, edges with weights between 0.01 and 0.9 (blue) represent a more diversified and flexible linkage pattern. Complementing this, Fig. 3b shows the network consisting only of extremely low-weight edges (weight <0.01). This part of the network is remarkably sparse, capturing transient co-occurrences that likely reflect strain-specific rearrangements or stochastic proximity events.

The structural backbone highlighted in Fig. 3a reveals distinct genomic communities. It is important to note that these communities do not necessarily correspond to functional operons (Fig. S3); rather, they represent regions in which the linear order of core genes remains highly resistant to rearrangement across the population. The fragmentation of the network into discrete communities suggests that the syntenic continuity of the core genome is not absolute but is frequently interrupted or truncated by the insertion of non-core genetic elements (e.g., genomic islands or mobile genetic elements) between conserved blocks. By integrating these high- and low-frequency co-occurrence patterns, the network provides a holistic view of the *E. coli* core genome’s spatial architecture. This analysis demonstrates that the core genome is organized into highly stable structural modules, partitioned by plastic non-core regions, offering a new perspective on how evolutionary pressures balance the preservation of core gene order with the dynamic influx of accessory genetic material.

We further quantified the distribution of edge weights across the entire core gene synteny network. The results reveal a distinct bimodal distribution, with weights concentrated near 0.01 and 1.0 (Fig. S4). This polarization underscores the coexistence of an ultra-stable structural backbone (high-weight edges) and highly variable genomic regions that are frequently interrupted by non-core elements. Beyond edge weights, the topological features of network nodes—specifically their degree distribution—offer critical insights into the evolutionary dynamics of gene families (Fig. S4). While the vast majority of nodes (*n* = 2,854) have a degree of 2, indicating stable linear synteny blocks, atypical node degrees highlight specific evolutionary events. Specifically, nodes with a degree of 4 (*n* = 7) indicate gene duplication events, in which duplicated paralogs are collapsed into a single orthogroup node that bridges two independent linear genomic segments. Conversely, nodes with degree 3 (*n* = 19) likely reflect lineage-specific gene loss, in which the absence of a gene in certain evolutionary branches produces a three-edge, branched connectivity pattern. Collectively, these topological signatures enable high-resolution inference of gene family expansion and contraction within the *E. coli* population.

**Fig 4:**
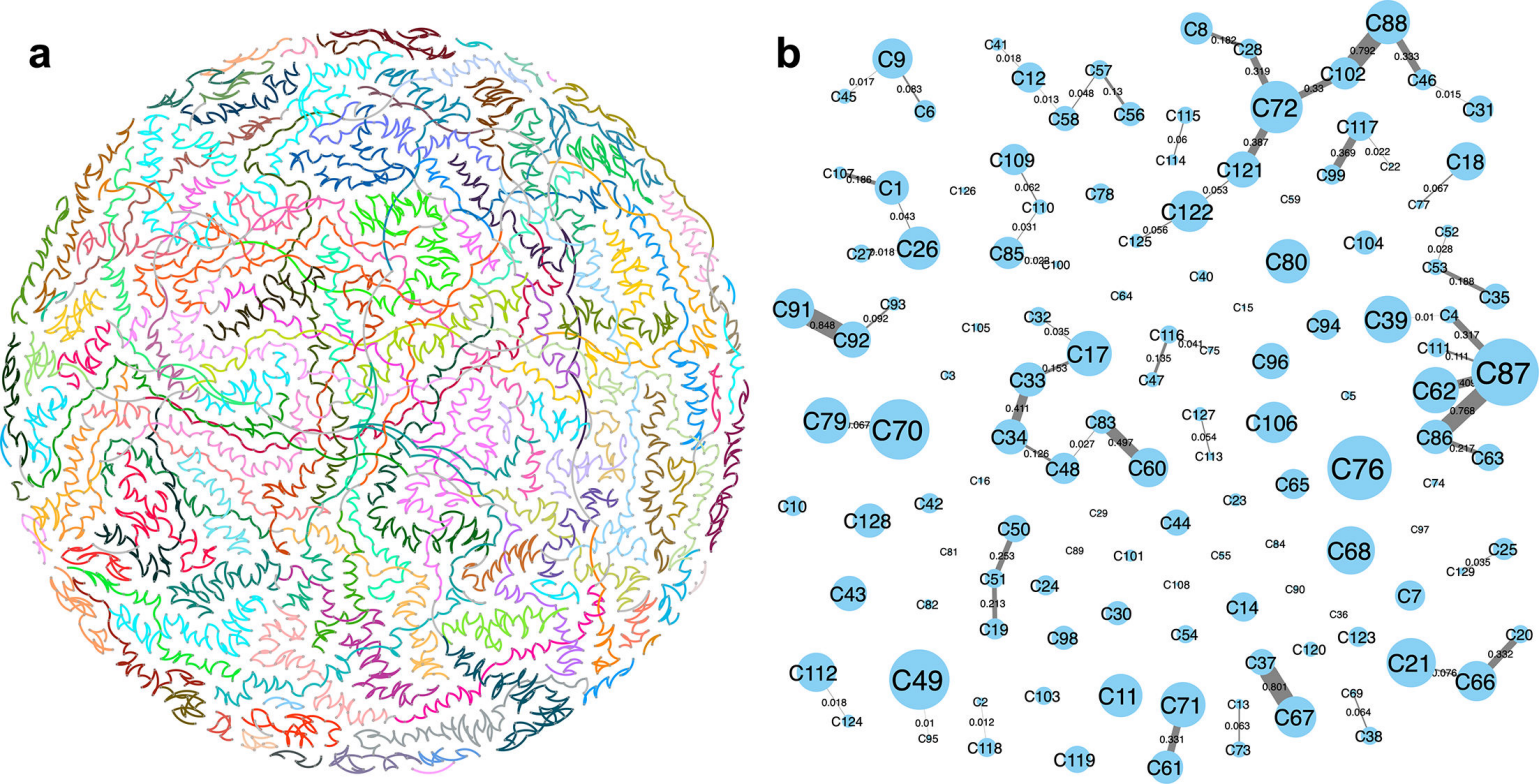
Modular community structure and inter-community relationships. (**a**) Community detection in the core synteny network. Different colors represent distinct communities identified by the Louvain algorithm (modularity *Q* = 0.9851). Each community corresponds to a stable genomic block. (**b**) Community association network. A coarse-grained representation where nodes represent entire communities, and edges reflect the total inter-community connection strengths. Node sizes and edge thicknesses are proportional to the number of orthogroups and inter-links, respectively.

To gain deeper insights into the functional and structural organization of core orthogroups, we performed community detection on the initial core orthogroups association network (Fig. 3a). Using the Louvain algorithm, we obtained a high modularity score of 0.9851, indicating a pronounced modular architecture with dense intra-community connections and relatively sparse inter-community links. In Fig. 4a, distinct colors represent the identified communities; these modules indicate genomic regions where the linear gene order is highly stable and evolutionarily conserved across the *E. coli* population. To further analyze the higher-order connectivity among these modules, we constructed a community association network (Fig. 4b) by collapsing each community into a single node, with edge weights computed as the sum of all original inter-community connections. This network revealed that while a subset of communities is tightly interconnected—suggesting potential functional coordination—the majority of modules appear isolated or only weakly linked. These inter-community connections are often interrupted by non-core genomic regions. Overall, the community structure clearly reflects a complex organizational pattern within the core genome, characterized by high structural modularity and significant independence among conserved blocks.

To further validate the phylogenetic consistency of the observed synteny architecture, we assigned each of the 1,200 complete *E. coli* genomes to a phylogroup using ClermonTyping. This method classified the genomes into nine major phylogroups (A, B1, B2, C, D, E, F, G, and clade I; see Table S5 for details). For each phylogroup, we independently reconstructed a core orthogroup synteny network from the same set of 3,053 core OGs. Community detection using the Louvain algorithm revealed that the phylogroup-specific networks exhibited a more fragmented yet more compact modular architecture compared with the global network built from all 1,200 genomes (Fig. S6; network structure information for each phylogroup is provided in Table S6. This pattern arises because the global network acts as a union that aggregates all possible syntenic connections across the entire population, whereas each phylogroup retains only lineage-prevalent connections. Consequently, inter-phylogroup variations in connectivity shape distinct community structures. To quantify these variations, we mapped phylogroup-specific and shared syntenic edges (Fig. S7). Together, these analyses underscore that although a common spatial backbone exists, the core genome synteny network is not uniform across phylogroups. Lineage-specific connections contribute to a more fragmented, tightly clustered local organization, and the union of all phylogroups recovers the full spectrum of possible gene adjacencies.

### Spatial distribution and genomic island association of non-core genes

**Fig 5:**
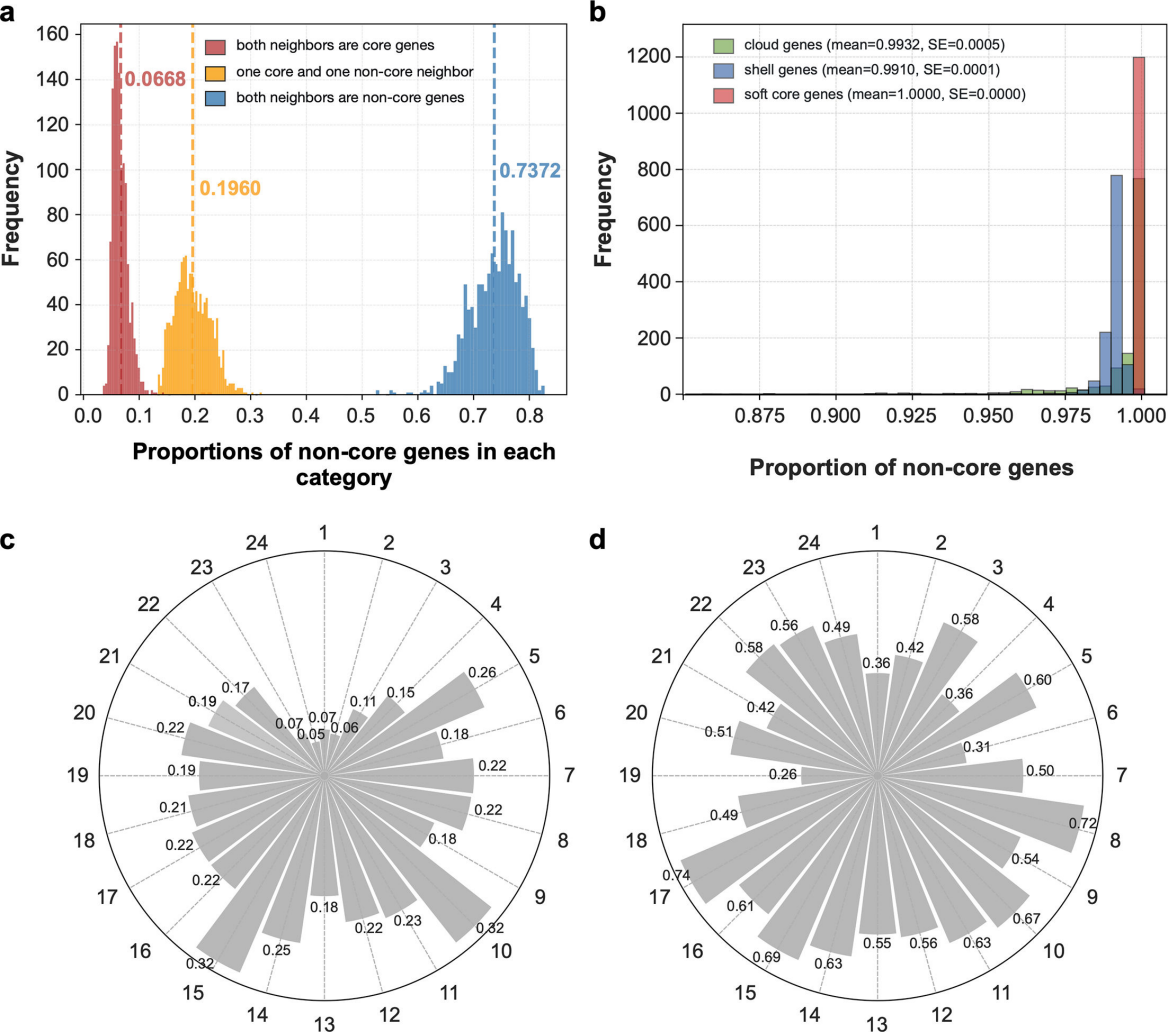
Non-core gene clustering and association with genomic islands. (**a**) Genomic neighborhood of non-core genes. Proportion of non-core genes flanked by core/non-core neighbors, highlighting the tendency of non-core genes to cluster (73.72% are flanked by other non-core genes). (**b**) Connectivity of flanking core genes. Distribution of edge weights for core orthogroups adjacent to non-core segments across different pangenome categories (soft core, shell, and cloud). (**c**) Spatial distribution of genomic islands (GIs). The proportion of GIs across 24 genomic intervals starting from the *oriC*. (**d**) Correlation between non-core clusters and GIs. Enrichment of GIs within extended non-core segments (>40 genes) compared to the genome-wide average across different chromosomal intervals.

We analyzed the flanking genes of each non-core gene across 1,200 complete genomes and classified their genomic contexts into three categories. Statistical results showed that non-core genes are predominantly flanked by other non-core genes on both sides (73.72%), whereas cases with one core neighbor (19.6%) or two core neighbors (6.68%) are significantly less frequent (Fig. 5a). This distribution pattern indicates that non-core genes are not randomly scattered but instead cluster into distinct genomic regions, such as genomic islands (53, 54). In contrast, the scenario in which a non-core gene is flanked by conserved core genes on both sides is relatively rare, suggesting that such genes may have unique functional or evolutionary contexts. Overall, these findings support the characteristic clustering of non-core genes in bacterial genomes, consistent with the emerging concept that accessory genes are not randomly distributed but exhibit deterministic co-occurrence relationships shaped by long-term selection (55, 56).

To further investigate their positioning within the association network, we assessed whether non-core genes reside in low-weight regions, based on the properties of their neighboring core genes. We defined a non-core gene as being in a low-weight region if its nearest flanking core genes belonged to orthogroups connected by edges with weights below 0.01 (blue edges in Fig. 3a). Across all conservation levels—soft core, shell, and cloud—the proportions of non-core genes in these low-weight regions were 100%, 99.1%, and 99.32%, respectively (Fig. 5b). This consistent pattern confirms that non-core genes are located in structurally loose and weakly linked genomic segments, suggesting they have greater evolutionary independence and plasticity.

The widespread distribution of these non-core regions is closely linked to the integration of GIs. By partitioning the chromosome into 24 intervals, we observed that the region surrounding the replication origin (*oriC*, interval 1) has the lowest GI proportion, likely due to stringent evolutionary constraints (Fig. 5c). Conversely, intervals 10 and 15 emerged as significant GI hotspots. Furthermore, extended non-core segments (containing >40 consecutive genes) showed a GI density substantially higher than the genome-wide average, particularly in intervals 8, 10, 15, and 17 (Fig. 5d). Collectively, these findings demonstrate that the *E. coli* pangenome is organized so that non-core genes and GIs are strategically sequestered in specific, structurally variable regions, preserving the integrity of the core structural backbone while facilitating niche-specific adaptation.

## DISCUSSION

Our analysis of 1,200 complete *Escherichia coli* genomes reveals that the species maintains its genomic identity not through random gene accretion but via a highly organized spatial architecture, best described as a structural backbone with sequestered plasticity. This model integrates two fundamental, interdependent principles: a rigid, modular, and asymmetrical scaffold formed by core genes, and the strategic confinement of accessory genetic elements—including genomic islands and other non-core regions—to specific integration hotspots defined by weak syntenic linkages within that scaffold. This spatial organization, revealed by combining high-throughput, alignment-free orthology inference (CVNet) with genome-wide synteny network analysis, provides a novel resolution to the long-standing paradox of how bacterial genomes reconcile evolutionary stability with the extensive genomic flux inherent in an open pangenome. It demonstrates that stability and plasticity are not opposing forces distributed randomly but are physically compartmentalized features of the chromosome, each underpinned by distinct topological rules.

The structural backbone—composed of tightly linked, evolutionarily recalcitrant core gene blocks—exhibits pronounced asymmetry and a striking bias toward the replication origin (*oriC*). This is not merely a statistical artifact but likely reflects a multi-layered adaptive optimization. Indeed, most bacterial gene families are subject to natural selection at specific chromosomal positions; this selection broadly shapes gene positioning to support variation in relative gene dosage in response to transient bacterial growth rates, a mechanism that affects most gene families. In this context, the concentration of *E. coli* core gene blocks toward *oriC* can be viewed as a special case of this general principle (52). First, clustering essential genes near the *oriC* maximizes their copy number during rapid growth, providing a dosage advantage for central metabolism and translation. Second, the conserved, modular order within these blocks may minimize disruptive collisions between the replication and transcription machineries that traverse these most active genomic regions. Most significantly, the high modularity (*Q* = 0.9851) of the backbone indicates that its stability is not monolithic but segmented. These modules likely represent fundamental units of chromosomal topology and co-regulation. However, the weak linkages between modules create natural, low-risk boundaries that tolerate interruption—thereby defining the very hotspots where plasticity is permitted. Thus, the backbone’s rigidity precisely defines the architectural context for controlled flexibility; its stability is not merely an endpoint but a prerequisite for the safe integration of novel genetic material.

Conversely, the sequestered plasticity of the accessory genome is not a passive consequence of exclusion but an active evolutionary strategy enabled by the backbone’s architecture. The finding that over 99% of non-core genes reside in genomic intervals flanked by core orthogroups with extremely low edge weights (<0.01) indicates that these regions are permissive integration hotspots. These hotspots likely experience relaxed structural and topological constraints, resulting in lower fitness costs for insertions that might otherwise disrupt essential gene clusters or local chromatin organization. This strategic compartmentalization offers a formidable solution to the dilemma posed by HGT; it allows a genome to rapidly sample a vast pool of adaptive functions from the environmental metagenome while physically insulating these experimental additions from the core operational machinery. The GIs enriched within these hotspots exemplify this principle, serving as modular “plug-and-play” cassettes for niche-specific adaptation. Thus, the backbone does not merely resist change; it actively channels and buffers genetic innovation into designated zones, transforming potentially disruptive HGT into a structured and manageable driver of evolution.

Collectively, these insights coalesce into a “modular backbone-hotspot integration” framework for understanding bacterial genome architecture and evolution. This framework shifts the perspective from a static catalog of gene content (the “bag of genes”) to a dynamic, spatially resolved view of how genetic material is organized, maintained, and renewed. It posits that the evolutionary trajectory of a bacterial species is shaped not only by the gain and loss of individual genes but, crucially, by the evolution of its genomic blueprint—the specific arrangement of stable modules and the permittivity of the inter-modular spaces that accept new genes. A key prediction of this model is that the degree of backbone rigidity and the number, size, and selectivity of integration hotspots should vary across species, forming a continuum that reflects their distinct ecological lifestyles and evolutionary strategies. For instance, pathogens with highly dynamic accessory genomes might exhibit more numerous or leakier hotspots, whereas streamlined, free-living microbes might possess a more consolidated and rigid backbone. Testing this prediction across the bacterial domain, now feasible with tools like CVNet, will reveal whether the elegant compartmentalization observed in *E. coli* represents a universal paradigm or one particularly refined in organisms navigating complex, variable environments.

In conclusion, by integrating the CVNet pipeline for scalable, alignment-free orthology inference with genome-wide synteny network analysis, we have moved beyond gene catalogs to decipher the spatial logic of a bacterial pangenome. The resulting modular backbone-hotspot integration model explains how *E. coli*, and potentially many bacterial species, architecturally reconcile the opposing demands of evolutionary fidelity and adaptive innovation. Looking forward, this spatially explicit approach establishes a foundation for topological pangenomics. Immediate priorities include elucidating the cis-acting sequences or structural features that define integration hotspots, understanding how backbone modules are established and maintained over deep evolutionary time, and investigating whether disrupting this spatial organization compromises genomic fitness (for example, by forcing integration into core blocks). Furthermore, applying this framework to clinically or ecologically important cohorts will reveal how shifts in backbone rigidity or hotspot usage drive pathogen adaptation, the dissemination of antibiotic resistance, or niche specialization. Ultimately, viewing genomes not just as lists of genes but as structured, evolving topological maps, as enabled by this study, will provide a deeper synthesis of genomics, evolution, and cellular design.

## MATERIALS AND METHODS

### The CVNet framework and orthogroup identification

Orthogroups were identified using CVNet, an alignment-free software developed in this study. The methodology encompasses four integrated stages:

- *K*-mer decomposition and vector construction: Gene sequences were decomposed into overlapping substrings of fixed length (*k*-mers). To effectively distinguish evolutionary signals from stochastic background noise, we implemented the Hao method, which uses a ()-order Markov model for background correction. Alternatively, the Count method was used to map raw *k*-mer frequencies. The resulting normalized value for each *k*-mer serves as a vector component, and components are explicitly set to zero when the corrected frequency is zero or negative. Finally, all components, each corresponding to a unique *k*-mer, are arranged in a fixed lexicographical order to form the gene’s high-dimensional composition vector, with the total dimensionality. This subtraction process enhances the significance of strings that contribute to amorphous characteristics.
- Similarity matrix calculation: Pairwise similarity between composition vectors was quantified using a suite of metrics, including Cosine similarity, InterList similarity, Jaccard index, and Dice coefficient, providing flexibility across different biological contexts.
- Sequence similarity network construction: The similarity matrix was transformed into a gene association network using three distinct strategies: RBH for high-confidence orthology; CUT for threshold-based filtering; and GBR, a hybrid approach that determines a global threshold based on the minimum reciprocal best hit across all genomes.
- Sequence clustering: Finally, the MCL algorithm was employed to partition the association network into discrete orthogroups. The inflation parameter was fine-tuned (e.g., between 1.5 and 3.0) to optimize clustering granularity.

To evaluate CVNet’s performance, we selected four widely used orthology inference tools for comparison: Roary v3.13.0 (15), PGAP2 v1.0.6 (17), OrthoFinder v2.5.5, and OrthoFinder v3.1.0 (57). Among them, Roary relies on conserved gene neighborhood information and runs relatively fast; OrthoFinder2, one of the most commonly used tools, generally offers high inference accuracy; OrthoFinder3 achieves incremental ortholog assignment for large data sets based on the core algorithm of OrthoFinder2; PGAP2, a more recent method, maintains fast execution while ensuring good accuracy.

### Data acquisition and preprocessing

To construct a high-quality, representative data set for downstream analysis, we initially downloaded 6,506 complete *Escherichia coli* genomes from the NCBI database. To ensure species consistency and exclude potential atypical strains, we performed average nucleotide identity (ANI) comparisons of all genomes against the *E. coli* type strain (GCA_003697165.2) using fastANI v1.165 (58), and applied a stringent 95% ANI threshold for filtering. This step removed 18 genomes with ANI values below 95%, leaving a final set of 6,489 high-quality *E. coli* genomes (Table S1). All test data sets used for subsequent CVNet-based analyses in this study were randomly sampled from this refined collection. To systematically assess the genetic diversity structure of this collection and establish a manageable yet representative subset for in-depth analysis, we plotted a histogram of the ANI distribution with a bin width of 0.2 (Fig. S8). Based on this distribution profile, we employed a stratified sampling strategy to select 1,200 genomes. We used ClermonTyping to assign the 1,200 genomes to phylogroups. The results are detailed in Table S5. This subset was designed to optimally capture the overall genetic diversity of the original data set and was used for subsequent pangenome analysis of *E. coli*.

### Determination of the origin of chromosomal replication

The origin of chromosomal replication (*oriC*) was predicted for each *E. coli* genome by combining two approaches: DnaA box enrichment and the position of the *dnaA* gene. In *E. coli*, the *oriC* contains five DnaA boxes (R1–R5), AT-rich 13-mer repeats, and regulatory protein-binding sites (59, 60). Although GC skew asymmetry is a common predictor of bacterial replication origins, we primarily relied on two principles: (i) DnaA boxes—the 9-bp consensus motif (TTATCCACA)—are enriched near *oriC*; and (ii) the *dnaA* gene is typically adjacent to *oriC*, with its transcriptional orientation matching the direction of replication (61). For most genomes, *dnaA* and *dnaN* are adjacent; we defined *oriC* as the intergenic region between them, where DnaA boxes are concentrated. When genome annotation was ambiguous, or the *dnaA-dnaN* adjacency was absent, we localized *oriC* by scanning for DnaA box enrichment in a 10 kb window upstream of *dnaA*.

### Core gene synteny network and community analysis

We constructed a core orthogroup synteny network based on the chromosomal adjacency of 3,053 core orthogroups across 1,200 genomes. In this network, nodes represent orthogroups, and edge weights represent the normalized frequency of gene-pair adjacencies. The Louvain algorithm was applied to the network to identify modular communities (62). The algorithm optimizes the modularity (*Q*) score to partition the core genome into densely interconnected blocks that exhibit high spatial stability. Edge weight distributions and node degrees were quantified using the NetworkX library (63). Atypical node degrees (e.g., degrees 3 or 4) were systematically identified to infer putative gene loss or duplication events. Both the core gene synteny network (Fig. 3) and the community connectivity network (Fig. 4a) were visualized and analyzed in Gephi, using a combination of the Force Atlas and Fruchterman-Reingold layout algorithms for spatial arrangement.

### Functional annotation and enrichment analysis

To systematically characterize the functional profiles of core and non-core orthogroups, we performed functional annotation of all orthogroups using the eggNOG database (64). Based on these annotations, COG category distribution plots for core and non-core orthogroups were generated in Python, enabling a visual comparison of their functional composition across major biological categories. Subsequently, to investigate the functional specificity of each detected community in the association network, we extracted gene sets from individual communities and performed pathway and functional enrichment analysis using KOBAS (65). Finally, based on the significant enrichment results from KOBAS, a functional enrichment heatmap was constructed to visualize the degree of enrichment of each community across specific biological pathways or functional terms.

### Genomic island prediction and spatial mapping

To investigate the spatial relationship between non-core gene regions and mobile genetic elements, we identified GIs in all 1,200 genomes using IslandPath-DIMOB v1.0.0 (66), which detects GIs based on sequence composition differences and the presence of mobility-related features. The *E. coli* chromosome was divided into 24 equal-length intervals from the replication origin (*oriC*) to analyze the distribution of non-core genes and GIs. The statistical significance of the asymmetric gene distribution was evaluated using the Kolmogorov-Smirnov test.

## Data Availability

The source code of CVNet is written in C ++ and available on GitHub (https://github.com/ghzuo/cvnet) under the MIT license.

## SUPPLEMENTAL MATERIAL

Supplemental material msystems.00207-26-s0001.docx Supplemental table captions and figures.

Supplemental tables msystems.00207-26-s0002.xlsx Tables S1 to S6.

ASM does not own the copyrights to Supplemental Material that may be linked to, or accessed through, an article. The authors have granted ASM a non-exclusive, world-wide license to publish the Supplemental Material files. Please contact the corresponding author directly for reuse.
