# Supplementary material for: A structural backbone with sequestered plasticity organizes the Escherichia coli pangenome
Source: mSystems. 2026 Jun 15;11(7):e00207-26. doi: 10.1128/msystems.00207-26 (PMC13386857; doi:10.1128/msystems.00207-26)

**Supplementary Materials for**

**A structural backbone with sequestered plasticity organizes the *Escherichia coli* pangenome**

Yi-Fei Lu^1^, Guang-Hong Zuo^2,*^, Xiao-Yang Zhi^1,*^

^1^ Yunnan Institute of Microbiology, Key Laboratory of Microbial Diversity in Southwest China of Ministry of Education, School of Life Sciences, Yunnan University, Kunming 650091, China

^2^ Wenzhou Institute, University of Chinese Academy of Sciences, Wenzhou 325001, China

* Authors for correspondence: Guang-Hong Zuo, ghzuo@ucas.ac.cn; Xiao-Yang Zhi, xyzhi@ynu.edu.cn

**Supplementary tables**

All supplementary tables are organized in a Microsoft Excel file named supplementary_tables.xlsx.

**Supplementary Table 1:** **Complete genome dataset of *Escherichia coli*.**

**Supplementary Table 2: Evaluation of different algorithmic parameters on clustering performance (with other parameters kept at default values).**

**Supplementary Table 3:** **Complete genome dataset of *Staphylococcus aureus*.**

**Supplementary Table 4:** **Complete genome dataset of *Klebsiella pneumonia*.**

**Supplementary Table 5: Summary of the Escherichia coli genome dataset evaluated in the pan-genome analysis.**

**Supplementary Table 6: Topological properties of *Escherichia coli* synteny networks across different phylogroups.**

**Supplementary Fig. 1: Sequence conservation and variation dynamics within *Escherichia coli* orthogroups.** a. Frequency distribution of mean sequence similarity within orthogroups. b. Frequency distribution of sequence variance within orthogroups. c. Linear correlation analysis between sequence variance and mean identity across orthogroups, with the red dashed line representing the linear regression fit.


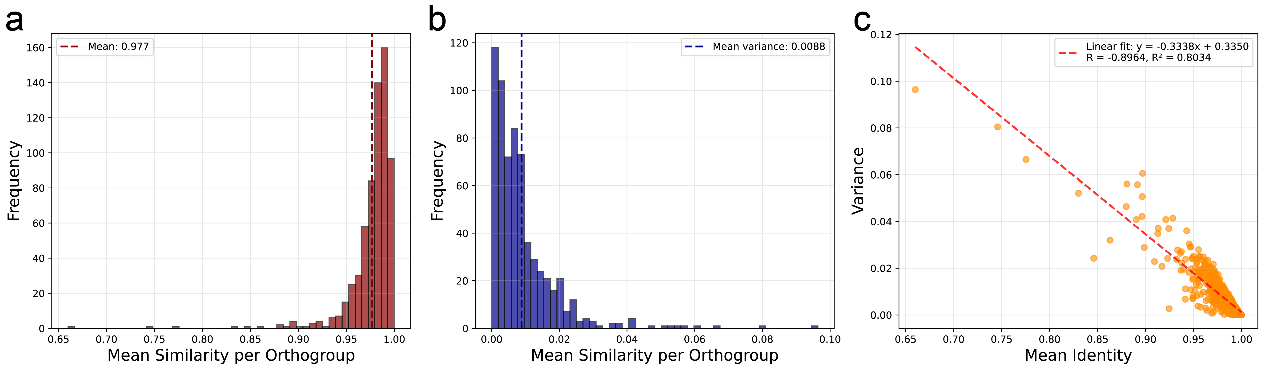


**Supplementary Fig. 2: Functional profiles of *Escherichia coli* core and cloud pangenome components.** Comparative COG (Clusters of Orthologous Groups) functional classification between core genes (present in ≥ 99% of strains) and cloud genes. Functional annotations were derived via eggNOG, with category S (Function unknown) excluded from the analysis.

**
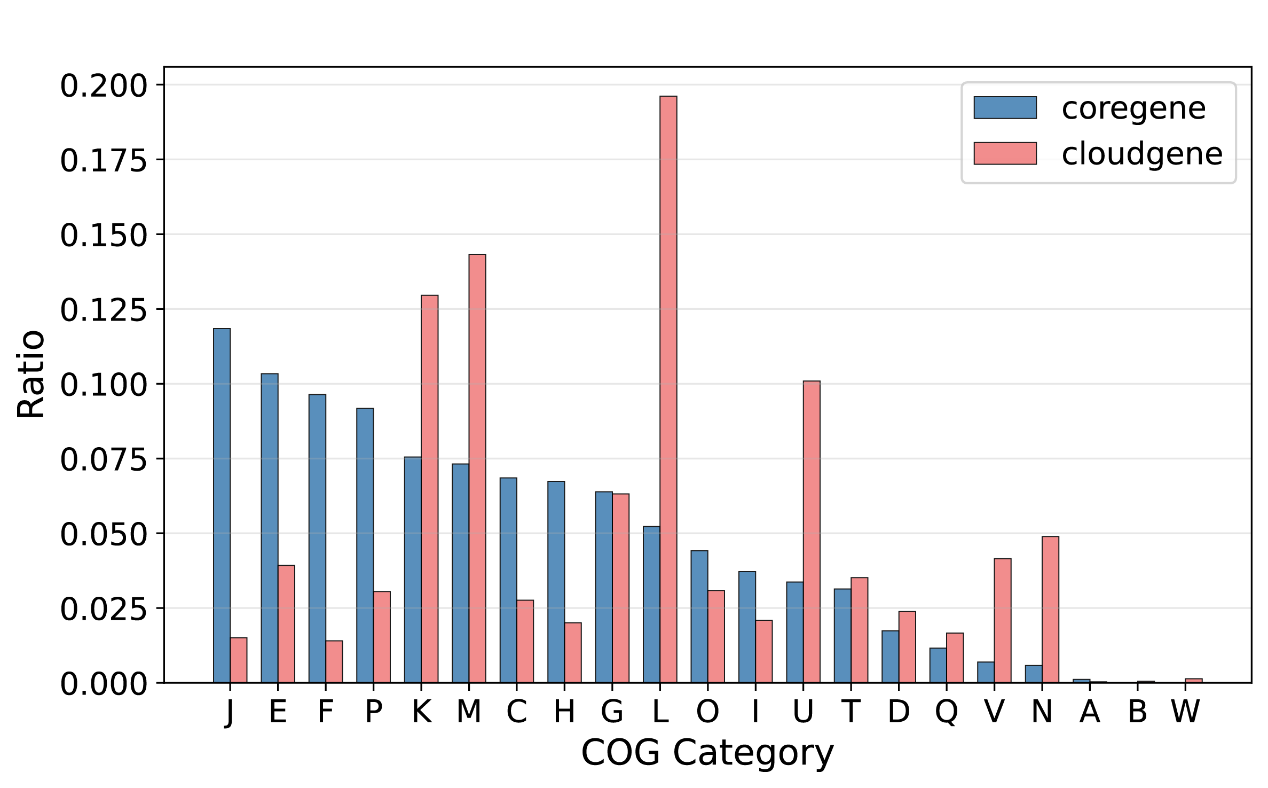
**

**Supplementary Fig. 3: KEGG pathway enrichment patterns across core orthogroup communities.** Heatmap displaying functional enrichment scores for individual communities. The upper panel delineates major metabolic pathways, while the lower panel indicates auxiliary pathways. Blue frames highlight statistically significant enrichments with *p*-values <0.05.

**Supplementary Fig. 4: Edge weight distribution within the core orthogroup synteny network**. Probability density function (PDF) curve coupled with a probability histogram demonstrating the distribution of normalized edge weights, where the horizontal axis represents the number of occurrences scaled by a factor of 1/1200.


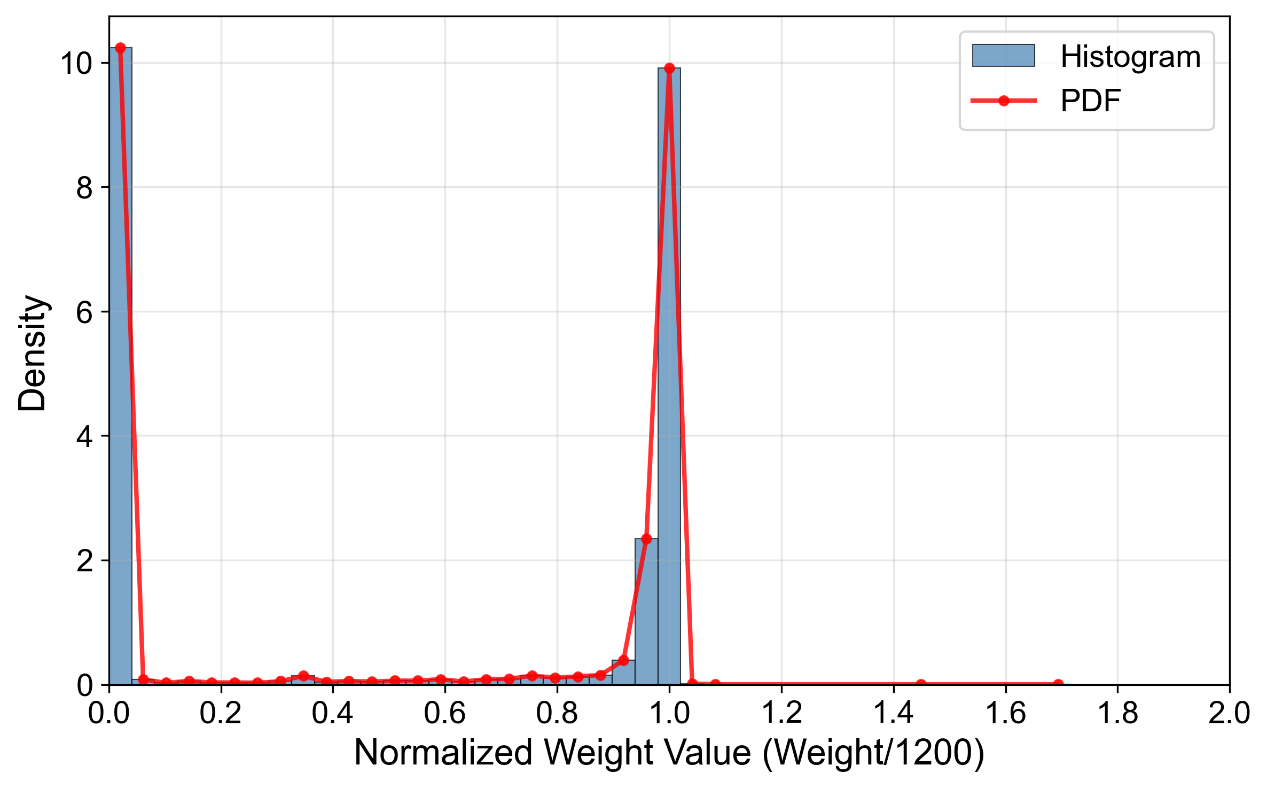


**Supplementary Fig. 5: Topological degree configuration and associated evolutionary events in the core synteny network.** Topological structure and node degree statistics of the core orthogroup synteny network. Variations in node degree correspond to specific evolutionary dynamics; for instance, a node with degree 4 typifies a gene duplication event, whereas a degree of 3 indicates a subsequent orthogroup loss following duplication.


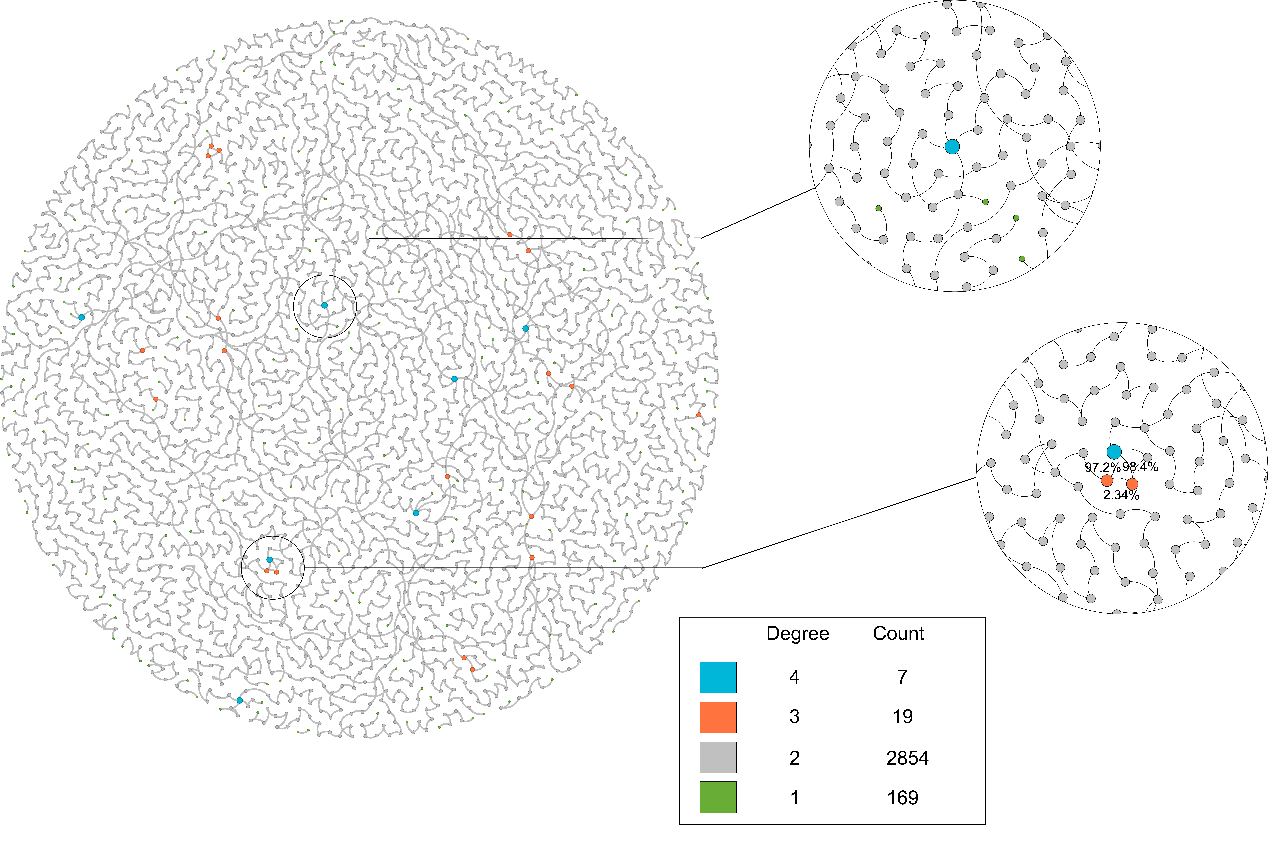


**Supplementary Fig. 6: Variations in synteny network community diameters across different *Escherichia coli* phylogroups.** Box plot highlighting the distribution of community diameters (measured in hops) within the synteny networks of major phylogroups.


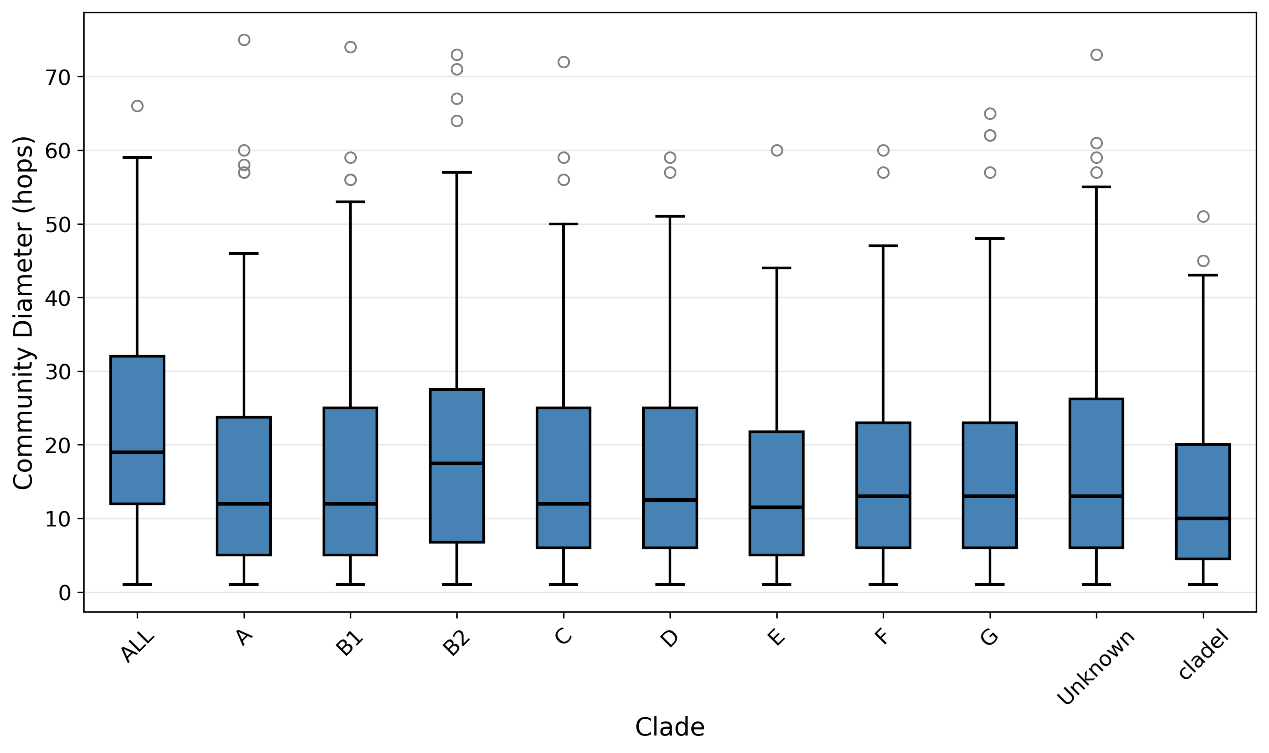


**Supplementary Fig. 7: Cross-phylogroup distribution matrix of unique and shared syntenic links.** Heatmap matrix displaying syntenic link counts across distinct *E. coli* network types. Diagonal cells represent unique links restricted to a single phylogroup, while off-diagonal elements indicate links shared between different lineages.
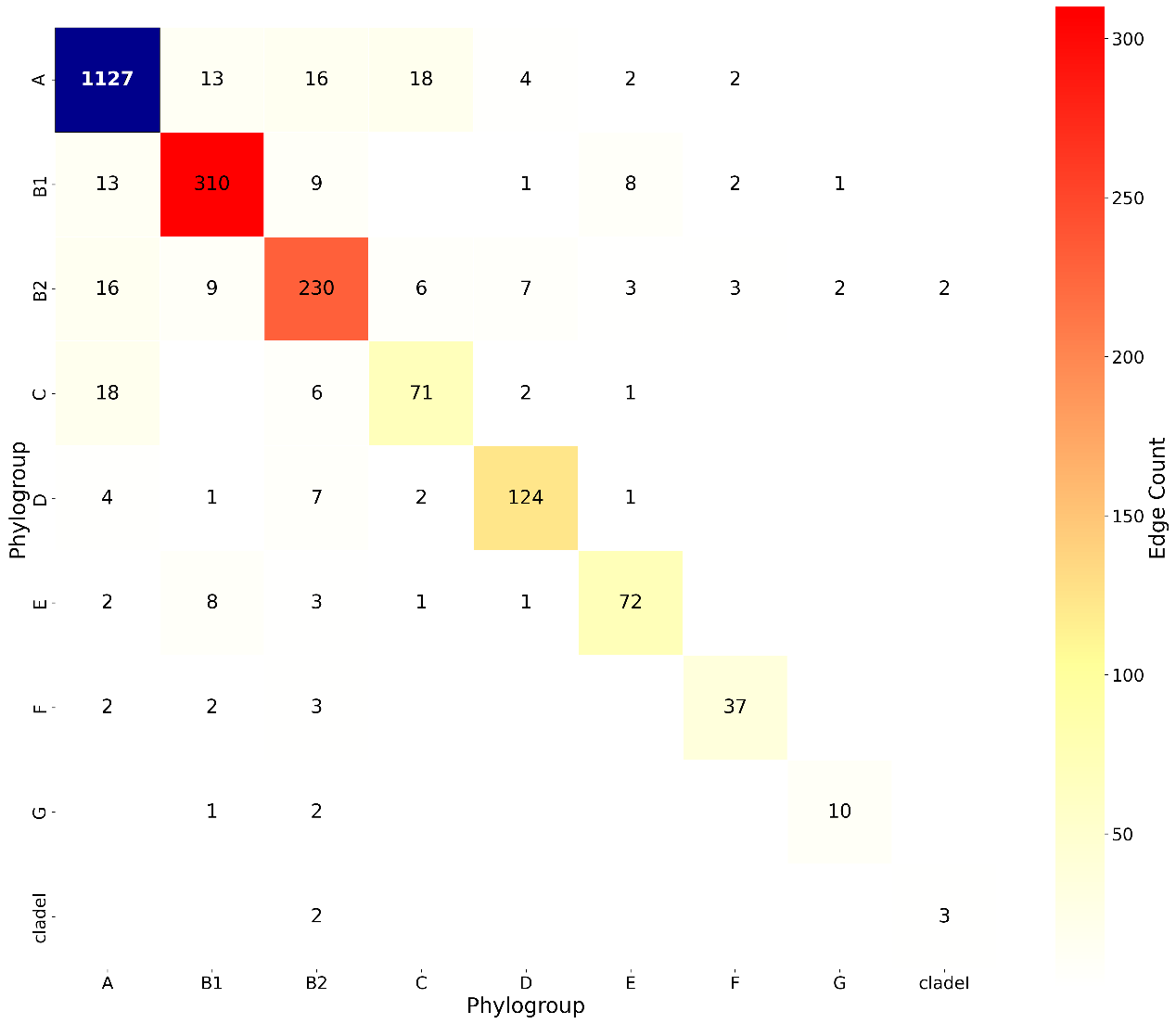


**Supplementary Fig. 8: Genomic divergence profile (ANI) of complete *Escherichia coli* genomes relative to the type strain.** Frequency distribution of Average Nucleotide Identity (ANI) values against the *E. coli* type strain (NCBI Accession: GCA_003697165.2). Stratified sampling was performed using an ANI bin size of 0.2.


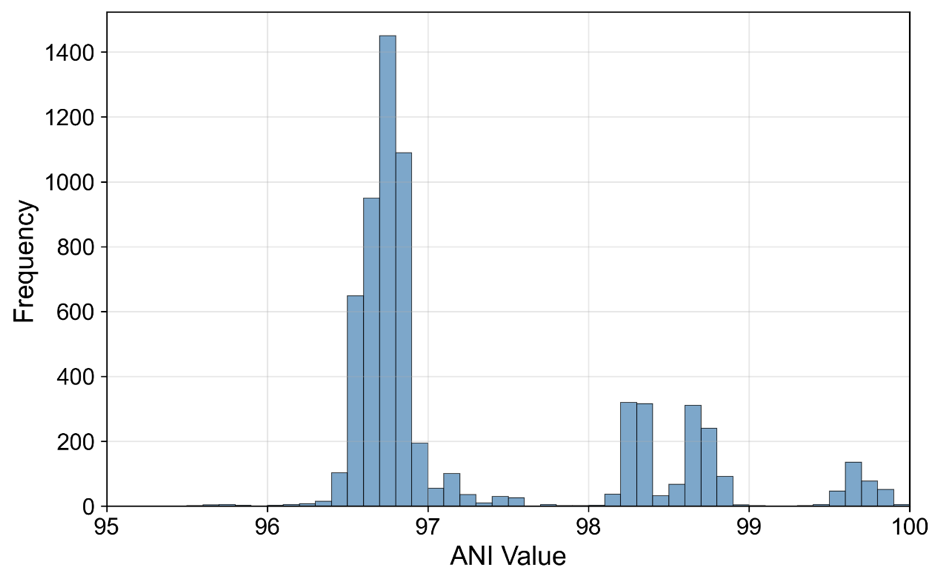

Supplement: Supplemental material — Supplemental table captions and figures. [file msystems.00207-26-s0001.docx]
